# The Current Management of Cardiac Tumours: a Comprehensive Literature Review

**DOI:** 10.21470/1678-9741-2019-0199

**Published:** 2020

**Authors:** Mihika Joshi, Siddhant Kumar, Arish Noshirwani, Amer Harky

**Affiliations:** 1 Countess of Chester Hospital, Chester, United Kingdom.; 2 Aintree University Hospital, Liverpool, United Kingdom.; 3 Department of Cardiothoracic Surgery, Liverpool Heart and Chest Hospital, Liverpool, United Kingdom.

**Keywords:** Heart Neoplasms, Publications, Research, PubMed

## Abstract

**Objective:**

To understand the current evidence and guidelines behind the appropriate management of cardiac tumours.

**Methods:**

A comprehensive electronic literature search has been performed in major databases - PubMed, Embase, Scopus, Ovid, and Google Scholar. All articles that discussed all different forms of cardiac tumours, their clinical presentation, diagnosis, and management methods have been critically appraised in this narrative review.

**Results:**

All relevant studies have been summarized in appropriate sections within our review. Cardiac tumours are rare but can be catastrophic and life-threatening if not identified and managed on timely manner. Utilization of all the available imaging methods can be of equivocal importance, relevant to each cardiac tumour. Surgical excision is the ultimate treatment method, however histopathological results can guide the adjunct treatment.

**Conclusion:**

Early detection of cardiac tumours has significant effect on planning the method of intervention. Technological advancements and increased availability of imaging modalities have enabled earlier and more accurate detection of these tumours. Novel medical therapies, recommendations for screening, and operative techniques have all contributed to overall improving knowledge of these tumours and ultimately patient outcomes.

**Table:** 

Abbreviations, acronyms & symbols	
3D	= Three-dimensional
AIDS	= Acquired immunodeficiency syndrome
AV	= Atrioventricular
C-CT	= Cardiac computed tomography
C-MRI	= Cardiac magnetic resonance imaging
CT	= Computed tomography
ECG	= Electrocardiogram
FDG-PET	= Fluourodeoxyglucose-positron emission tomography
HHV-8	= Human herpes virus 8
HMCM	= Hamartoma of mature cardiac myocytes
LHAS	= Lipomatous hypertrophy of the atrial septum
MDCT	= Multidetector computed tomography
TOE	= Transoesophageal echocardiography
TTE	= Transthoracic echocardiography
WHO	= World Health Organization

## INTRODUCTION

The incidence and prevalence of cardiac tumours remain one of the lowest amongst all tumours. The estimated prevalence of cardiac tumours is 0.001-0.03%^[1]^, whereas metastatic involvement of the heart is far commoner, with an estimated prevalence between 2.3-18.3%^[1,2]^.

Cardiac masses can be classified into either neoplastic or non-neoplastic masses. Neoplasms are abnormal tissue growths, which can be clinically characterized as either benign or malignant^[2]^. These growths can also be further stratified by the site of their origin; primary masses arise directly from cardiac tissue, whereas secondary masses migrate to cardiac tissue from peripheral sites. Whilst primary cardiac tumours can either be benign or malignant, secondary masses are always malignant, occurring in the presence of disseminated malignancy^[3]^. The latter is far commoner compared to primary cardiac neoplasms^[2]^.

Clinical presentation can vary from a commonly known triad of symptoms, including obstructive, embolic, or systemic symptoms, to asymptomatic presentations^[2,3]^. Symptoms can include, but are not limited to, congestive symptoms, such as orthopnea, dyspnoea, and frank haemoptysis as a result of florid pulmonary oedema; an embolic phenomenon, which can lead to acute pulmonary embolisms, strokes, or other cerebrovascular events; and systemic symptoms, including fevers, arthralgia, and rigors^[2-5]^.

Radiological imaging plays a key role in the diagnosis of cardiac tumours. Echocardiography, cardiac computed tomography (C-CT), and cardiac magnetic resonance imaging (C-MRI) are key imaging modalities utilized in this process^[6]^, with echocardiography being favoured given its noninvasive, accessible, and cost-effective nature^[5]^. More advanced imaging techniques such as fluourodeoxyglucose-positron emission tomography (FDG-PET) may be utilised to look for metastatic involvement of cardiac tumours or identify a primary source in the case of cardiac metastasis^[7,8]^.

Management of cardiac tumours greatly depends upon the tumours’ characteristics, location, and metastatic involvement. Surgical excision with or without the use of adjuvant therapies, such as chemotherapy or radiotherapy, remains the fundamental approach in the management of cardiac tumours^[2]^. In the case of aggressive disease, palliative treatment may be used with a goal to provide symptomatic relief rather than curative intent^[9]^.

## TUMOUR TYPES

Primary tumours are classified into benign or malignant tumours. Benign tumours comprise 75% of neoplasms affecting the heart^[9]^, encompassing myxomas, lipomas, fibroelastoma, rhabdomyoma, and fibromas. Comparatively, primary malignant cardiac tumours are rare and comprise 25% of all neoplasms. The main types of malignant neoplasms are undifferentiated pleomorphic sarcomas, which encompass angiosarcomas, rhabdomyosarcoma, leiomyosarcoma, liposarcoma, osteosarcoma, fibrosarcoma, and malignant fibrous histiocytoma^[2,9]^. Secondary cardiac tumours may result from systemic metastases and are associated most commonly with lymphoma, melanoma, and carcinomas of the thorax including breast, lung, and oesophagus^[2]^. Lastly, non-neoplastic masses such as lipomatous hypertrophy of the atrial septum (LHAS), thrombi, and foreign bodies make up a small proportion of cardiac masses, which can be considered as differentials, when investigating cardiac masses^[2]^.

In 2015^[10]^, the World Health Organization (WHO) updated the nomenclature of cardiac tumours, stratifying cardiac masses into either benign or malignant neoplasms^[10]^.

### Neoplastic Tumours

Neoplastic masses are subdivided into either primary or secondary tumours. Primary tumours arise directly from the cardiac cells and can be either benign or malignant.

### Benign Primary Neoplasms

#### Myxoma

Cardiac myxomas are the most common type of benign cardiac tumours arising from the endocardium of the heart, projecting into the cardiac chambers^[3,10]^. Most commonly arising from the left atrium, these tumours occur between the fourth and sixth decades of life and have a slight female predisposition^[2,4,9]^. Macroscopically, their average diameter is 6 cm, but they have been noted to be up to 15 cm in individuals and are attached via a pedicle to the cardiac wall^[9,11,12]^. Myxomas have an increased tendency to thromboembolise, which can lead to cerebral or coronary vascular embolisms^[2,3,10]^. The majority of myxomas are sporadic and unrelated to genetics or underlying conditions^[1-3,11]^. However, many authors have highlighted a link between recurrent myxomas and the Carney complex, an autosomal dominant endocrinopathy. Clinical presentation of myxomas depends vastly on location. Patients may present with embolic features, such as stroke or pulmonary embolism^[4]^, or may have obstructive and congestive symptoms, depending on whether the tumour is obstructing, the aortic or pulmonary outflow^[3,11]^. Conversely, patients may also be incidentally diagnosed upon presenting with systemic symptoms including weight loss, haemoptysis, anaemia, or long-term fevers^[2]^. Given the risks associated with embolic events, surgical resection is indicated for cardiac myxomas^[1,2]^.

#### Rhabdomyoma

A rhabdomyoma is a congenital hamartoma^[10]^, which is the most common benign cardiac neoplasm in the paediatric population and it mostly affects newborns, with an average age of two weeks at the time of diagnosis^[10,13]^. These neoplasms develop in utero and are often picked up with an ultrasound scan during prenatal screening. Rhabdomyomas often present with multiple lesions, usually developing in the ventricles or the ventricular septum of the fetal heart^[14]^. Clinical manifestation depends upon the age of presentation; it can include murmurs, cyanosis, respiratory distress, cardiac failure, and cardiogenic shock, along with hydrops fetalis and ventricular inflow/outflow obstructions, the latter two may be fatal for patients^[9]^. They may also be associated with tuberous sclerosis, with Hoffmeier et al.^[11]^ suggesting that approximately half the cardiac rhabdomyomas are linked to this genetic condition. Rhabdomyomas have been known to spontaneously regress, as noted by various authors^[2,3,10,13-15]^. Therefore, first line management of these masses is often conservative, and surgery is only considered if there are high-risk clinical presentations^[3,16]^.

### Other Rare Tumours

#### Fibroma

Fibromas are connective tissue neoplasms which occur mainly in childhood and have a slightly higher male predisposition^[3,10,17-19]^. They commonly arise from the interventricular septum or left ventricle and are categorised as an intramural or ventricular cardiac mass^[18,19]^. Clinically, fibromas may often present with ventricular arrhythmias given the most common tumour locations being the left ventricle and the interventricular septum. This can affect the conduction along the Bundle of His and likely result in such symptoms for the patients^[19]^. Further clinical manifestations include syncope, chest pain, and heart failure symptoms, but rarely it can present with ventricular arrhythmia and sudden death^[3,4,19]^. They are also associated with Gorlin syndrome, equally known as nevoid basal cell carcinoma, an autosomal dominant genetic condition due to mutations in the PCTH1 gene, which predisposes individuals to a wide range of developmental problems and a high likelihood for developing malignancies^[20]^.

#### Lipoma

Lipomas typically affect elderly women and have a higher incidence amongst individuals with increased body mass. They can be subendocardial, subpericardial, or endocardial and comprise encapsulated tumours composed of mature adipocytes^[2,3,11]^, differentiating them from LHAS tumours^[2,3,11,21,22]^. Incidentally detected, these tumours are best monitored through active surveillance, whilst symptomatic patients may be offered surgical resection^[2]^.

#### Cystic Tumour of the Atrioventricular Node

Atrioventricular (AV) node tumours are congenital endodermal multicystic lesions, arising at the base of the interatrial septa in the triangle of Koch^[2,22]^. They affect women more frequently than men (3:1), with a mean age of 38 years at presentation^[2]^. Given the integrity of the AV node in conduction physiology, presentation is most commonly with complete heart-block, with fewer still presenting with AV-block, and rarely sudden cardiac death^[23]^.

#### Hamartoma of Mature Cardiac Cells

Hamartoma of mature cardiac myocytes (HMCM) is a hyper-proliferative growth of mature cardiac cells, which tend to be slow growing, solitary lesions, and are histopathologically characterised by disorganised arrangement of hypertrophic cardiomyocytes^[24]^. It affects men twice as frequently as women, typically presenting in their mid-twenties^[2]^. Clinically, these tumours tend to have a predisposition to affect the ventricles and interventricular septum^[24]^ and tend to be asymptomatic. In some cases, the literature has noted HMCM to be associated with arrhythmias, intermittent chest pain, dyspnoea, and sudden death^[24,25]^. Table 1 is a summary of benign cardiac tumours.

**Table 1:** Benign cardiac tumours.

Tumour type	Clinical Features
Myxoma	Most common cardiac tumour, found mostly in the left atrium; female > male predisposition, 4^th^ to 6^th^ decades of life^[2,4,9]^ Systemic symptoms, including anorexia, heart failure, arrhythmias, dyspnoea, and syncope^[3,11]^ Embolic events: pulmonary or tumour embolism (high tendency to thromboembolise)^[4]^ Association with Carney complex - genetic mutation of PRKAR1A gene; symptoms of endocrine pathology, abnormal skin pigmentation, and extracardiac myxomas and endocrine hyperactivity may coexist^[1-3,9,11]^
Rhabdomyoma	Congenital hamartoma - most common benign paediatric cardiac tumour; average age at diagnosis »2 weeks^[10,14]^ Affects ventricles and ventricular septum, large range of sizes 0.3-9 cm^[1,14]^ Symptoms include murmurs, respiratory distress, ventricular inflow/outflow obstruction, and possible cardiogenic shock^[9]^ Association with tuberous sclerosis; mutations of TSC1 and TSC2 genes; diagnostic: triad of seizures, mental retardation, and facial angiofibromas^[1,2,11]^ Tumours may spontaneously regress
Fibroma	Associated with Gorlin syndrome, a genetic mutation of the PCTH1 gene^[21]^ Often found in ventricles, which results in arrhythmias; other symptoms include heart failure, obstruction of great vessels and outflow tracts, conduction abnormalities, chest pain, and syncope^[3,4,20]^
Lipoma	Can be subendocardial, subpericardial, or endocardial tumours^[3,11]^ Increased predisposition towards elderly women with increased body mass index^[2]^ Symptoms include obstructive symptoms, conduction abnormalities resulting in arrhythmias/AV block Conduction abnormalities^[2,3]^
Cystic tumour of the AV node	Also known as mesothelioma of the AV node^[2,24]^ Generally found at base of atrial septum, Koch's triangle^[23,24]^
Hamartoma of mature cardiac myocytes	Hyperproliferative, disorganised growth of mature cardiac myocytes Male > female predisposition, affected age » 20 years^[2]^ Commonly affects ventricles and interventricular septum Symptoms at presentation may include intermittent chest pain, dyspnoea, and, rarely, sudden death^[25,26]^

AV=atrioventricular

#### Malignant Primary Neoplasms

These tumours are relatively rare, with sarcomas representing 95% of such malignant neoplasms^[2,3]^. Other malignant tumours include undifferentiated pleomorphic sarcomas, lymphomas, and malignancies associated with Li-Fraumeni syndrome^[5]^. Table 2 is a summary of primary malignant cardiac tumours.

**Table 2:** Primary malignant cardiac tumours.

Tumour Type	Clinical Features
Angiosarcomas	Poor prognosis, male > female
	Commonly affects right atrium or ventricle^[2,3,19]^
	Symptoms presented late and include right-sided heart failure, cardiogenic shock, pericardial effusions
	Highly aggressive, with high rate of recurrence; echocardiography is first line imaging; C-CT and C-MRI are often used in metastatic disease involvement^[5,19,28]^
	Treatment is often palliative rather than curative
Rhabdomyosarcomas	Commonly affects either ventricle^[5]^
	Aggressive tumours with high rates of recurrence and metastatic involvement^[5,11]^
	Similar clinical presentation to rhabdomyomas; symptoms include ventricular outflow obstruction, cardiac failure, and cardiogenic shock^[4,5]^
Undifferentiated pleomorphic sarcomas	Most common malignant type of cardiac tumour; no gender predisposition, with mean age at presentation » 45 years^[2,29]^
	Commonly found in the left atrium, generally systemic presentation including symptoms such as dyspnoea, chest pain, and cough^[2,29]^
	Surgical resection is treatment of choice given patient suitability for surgery
Lymphoma	This malignancy is typically confined to the pericardium with minimal extracardiac involvement
	Male > female predisposition, affected age » 60 years^[31]^
	Increased incidence of lymphoma amongst immunosuppressed individuals (AIDS, HHV-8)^[2,31]^
	Symptoms can either be systemic, including nights sweats, malaise, and pyrexia, or cardiospecific, such as atrial fibrillation, heart failure, or pericardial effusions^[2]^
	Various treatment options, including chemo/radiotherapy, biological agents, such as monoclonal antibodies, and surgery (generally palliative intent)^[2,4,31]^
Li-Fraumeni Syndrome	Autosomal dominant inherited condition; associated with germline mutations of the TP53 gene
	Should be considered in young patients (< 45 years old), with recurrent cardiac tumour disease
	Increased predisposition of childhood cancers, most commonly associated malignancies, including sarcomas, brain tumours, breast cancer, leukemias, and adrenocortical cancers^[32,33]^

AIDS=acquired immunodeficiency syndrome; C-CT=cardiac computed tomography; C-MRI=cardiac magnetic resonance imaging; HHV-8=human herpes virus 8

### Sarcomas

#### Angiosarcomas

Though relatively uncommon tumours, angiosarcomas represent the most frequently occurring primary malignancies of the heart and have extremely poor prognosis associated with them^[2,3,18]^. These tumours have a higher predisposition in men compared with their female counterparts, with a suggested ratio of 2-3:1, respectively^[18]^. Angiosarcomas tend to affect the right atrium most commonly, with frequently occurring invasion into cardiac chambers and adjacent structures, such as the right pericardial wall^[10]^. This results in a multitude of symptoms depending on the tumour’s site, size, and involvement of adjacent structures.

Macroscopically, these tumours often have a haemorrhagic or necrotic appearance with a vascular channel lined with anaplastic, epithelial cells^[5,10]^. Microscopically, angiosarcomas are endothelial cell tumours with vascular anastomosing channels, with poorly defined areas of epithelioid and spindle cells^[18]^. The most common presenting symptoms include dyspnoea, right-sided heart failure, cardiogenic shock, and the development of a pericardial effusion^[26,27]^. Clinical manifestation of angiosarcomas is very late; with a large proportion of these tumours having already metastasized at diagnosis. Hence, these tumours confer a poor prognosis, with high rates of recurrence due to an aggressive tumour biology, a poor response to therapy, and a lack of targeted treatment^[18,27]^. Treatment is often directed with palliative, rather than curative intent, through partial or complete surgical resection and possible adjuvant chemotherapy^[5]^.

#### Rhabdomyosarcomas

These malignant neoplasms arise from the striated cardiac musculature^[11]^. They are the second most common form of malignant cardiac tumours and are associated with a higher rate of recurrence and metastasis^[3,5]^. They can arise from any cardiac chamber, but have a predisposition for the ventricles^[5]^. Studies have also described the highly aggressive nature of these tumours, with frequent invasion into valvular structures, the pericardium, pleura, mediastinum, and other distant organs^[5,11]^.

The WHO states that most cases occur in children or adolescents, with a mean incidence age of 14 years^[10]^. Microscopically, these tumours display pleomorphic nuclei embedded within an eosinophilic cytoplasm^[5]^. Clinically, rhabdomyosarcomas present in a similar manner to benign primary lesions, hence symptoms may be non-specific^[4]^.

#### Undifferentiated Pleomorphic Sarcoma

Undifferentiated pleomorphic sarcomas account for 10% of all primary malignant cardiac tumours and are known by numerous other names, including malignant fibrous histiocytoma^[2]^. Mean age at presentation is 45 years with both genders being equally affected^[28]^. These sarcomas are more commonly found in the left atrium and arise as endocardial growths which protrude into the chamber and adjacent myocardial tissue^[2,28]^. By definition, pleomorphic sarcomas lack specific morphological features of differentiation, however cells may express focal vascular or epithelial tumour markers, with MDM2 amplification being cited in literature as being found in a subset of these tumours^[2,28]^. Sarcomas present with non-specific symptoms such as dyspnoea, chest pain, and cough. Imaging with C-CT and later C-MRI demonstrates large irregular lesions as either infiltrating into myocardium, hemorrhagic pericardial mass, or noninvasive focal lesion causing obstructive symptoms^[2,28,29]^. Surgical resection is the treatment of choice in patients with acceptable performance status, with adjuvant chemoradiotherapy offered to those undergoing partial resection^[2,28]^.

### Other Rare Malignancies

#### Lymphoma

Primary cardiac lymphoma has limited or no extracardiac involvement. These malignancies are typically confined to pericardium and thought to arise from the epicardial lymphatic network^[2,4]^. Increased incidence of cardiac lymphoma has been associated with immunosuppression through organ transplantation, acquired immunodeficiency syndrome (AIDS), or infection with human herpes virus 8 (HHV-8) or Epstein-Barr virus^[2,15]^. Cardiac lymphoma is twice as common in men as in women, with most cases occurring in patients over the age of 60 years^[2,30]^. Patients present with constitutional symptoms of lymphoma, including pyrexia, malaise, night sweats, and weight loss, however with also specific conditions, such as atrial fibrillation, heart failure, and pericardial effusion^[2,4]^. Cardiac lymphomas span the range of B-cell lymphomas, including diffuse large B-cell, Burkitt’s, and low-grade^[10]^. Macroscopically, they appear as grey-white coalescent nodules occasionally extending from epicardium to myocardium^[4]^. Echocardiography most commonly identifies effusion, whilst C-CT and C-MRI are useful for staging purposes, with the latter demonstrating an extensive, homogenous, isointense mass on T1/T2-weighting with minimal gadolinium contrast uptake^[2,30]^. Treatment requires prompt histopathological diagnosis which guides specific management dependent on subtype, and these treatments are analogous to systemic lymphoma and include chemoradiotherapy and monoclonal antibody therapy^[2]^; surgical treatments are typically palliative to relieve obstruction^[30]^.

### Li-Fraumeni Syndrome

Li-Fraumeni syndrome is a rare, inherited autosomal dominant condition, which is associated with germline mutations in the TP53 gene^[31,32]^. This syndrome can be the aetiology for a minority of cardiac tumours, as suggested by Hoffeimeir et al.^[11]^, who recommended the determination of this diagnosis considering this as a differential in younger patients who present with recurrent cardiac tumour disease.

### Non-Neoplastic Masses

As described by Burke et al.^[10]^ in the new WHO classification, non-neoplastic masses include papillary fibroelastomas and LHAS. Each of these is further described below. Table 3 is a summary of non-neoplastic cardiac masses.

**Table 3:** Non-neoplastic cardiac masses.

Tumour Type	Clinical Features
Papillary fibroelastoma	Benign papillary endocardial growths, equal male to female prevalence^[2,9]^ Commonly located in the valvular endocardium - aortic and mitral valves^[1,16,34]^ High propensity to cause thromboembolic events, which may lead to cerebrovascular accidents and/or coronary occlusive events^[10,38]^
Lipomatous hypertrophy of the atrial septum	Commonly found in obese patients, higher female predisposition^[3,39]^ Common clinical manifestations, including obstructive symptoms, which may lead to congestive cardiac failure and arrhythmias^[10,40]^ Often asymptomatic, found on echocardiography; characteristic "dumb-bell" like appearance on echo^[2]^

### Papillary Fibroelastoma

Often known as endocardial papillomas, these benign endocardial papillary growths are most commonly found in the elderly with equal prevalence in males and females^[2,9]^. Most commonly affecting the mural endocardium of aortic and mitral valvular endocardium, they may also be found in non-valvular endocardial regions, such as the mural endocardium of the left ventricle^[1,15,33]^. These masses have a high predisposition to thromboembolise^[1,34,35]^, hence clinical presentation may often be in the form of cerebral or coronary occlusive events^[4]^. Echocardiography remains a key imaging modality^[36]^. Given the increased propensity to thromboembolise, robotic or traditional surgical resection with valvular sparing should be offered to all patients who are suitable for intervention^[10,37]^.

### Lipomatous Hypertrophy of the Atrial Septum

LHAS is a non-neoplastic cardiac pathology which predominates in obese patients and more commonly affects the elderly, female patients, as noted by Shapiro et al. and Bielicki et al.^[3,38,39]^. LHAS is classified as hypertrophy of the atrial septum and may manifest as obstructive symptoms and arrhythmias^[10,39]^.

The majority of patients remain asymptomatic with LHAS^[2]^, and this diagnosis is often made incidentally on imaging. LHAS has a characteristic “dumbbell-like appearance”, which is often seen on echocardiography^[2,38]^.

### Cardiac Metastasis

With an incidence of up to 10% in patients with malignancies^[11]^, cardiac metastases are far commoner compared to primary cardiac tumours, with the common sites of deposits involving the pericardium, myocardium, or endocardium^[3]^. Lymphomas, leukaemias, and melanomas have a higher rate of metastasizing to the heart than other malignancies via haematogenous spread^[2,4]^, whereas other malignancies spread via direct extension or lymphogenous spread, such as breast, oesophageal, or lung carcinomas^[2]^. Lastly, abdominal malignancies including renal and hepatocellular carcinomas can have metastatic involvement via movement through the inferior vena cava^[9]^.

Clinical manifestations and management of cardiac metastasis depend on anatomical, structural, and positional aspects of the metastasis, but often include the pericardium, leading to either pericardial effusions or cardiac tamponade as a result of the latter^[2,9]^.

## MODE OF PRESENTATION

Clinical presentation of cardiac tumours is variable. The symptoms depend on multiple factors including size, location, and infiltration of the tumour mass into adjacent tissues, and are less commonly affected by the tumour’s histological characteristics^[11,40]^. The triad of symptoms associated with cardiac tumours includes obstructive, embolic, and systemic symptoms; each of which is further discussed below. Conversely, tumours may often be asymptomatic, with these masses being detected incidentally upon clinical investigations. Metastasis to the heart often presents with symptoms of pericardial involvement and other systemic symptoms, as discussed below^[40,41]^.

### Obstructive Symptoms

Hoffmeier et al.^[11]^ have suggested that tumours in the region of AV valves may result in symptoms suggestive of valvular stenosis. Both Shapiro et al.^[3]^ and Hoffmeier et al.^[11]^ have also further concurred that compression of these valves may result in obstructive symptoms and can be largely related to body posture. Infiltration of the cardiac masses into the myocardium may cause symptoms of cardiomyopathies and result in symptoms of congestive cardiac failure, including dyspnoea, orthopnoea, and frank pulmonary oedema^[4,11]^.

### Embolic Phenomenon

Emboli can occur as a result of part of the tumour dislodging itself, or thrombi which surround or lie adjacent to the tumour^[3]^. Right-sided tumours normally embolise to the lung and present with features of a pulmonary embolism, whilst left-sided tumours may dislodge into the systemic circulation and lead to cerebrovascular events^[3]^. Hoffmeier et al.^[11]^ recommended performing histopathological analysis of all fragments from emboli to confirm its aetiology and potential source^[11]^.

### Systemic Manifestations

Apart from cardiac-specific symptoms, tumours may also be associated with systemic manifestations of malignancy, including weight loss, anorexia, arthralgia, and pyrexia^[4,41]^. Often, such features would prompt further investigations for primary malignancies, and cardiac masses may be picked up coincidentally. Moreover, these symptoms may often be also associated with infective processes, such as infective endocarditis, and, therefore, it is extremely important to perform appropriate imaging, which will allow differentiation between cardiac masses^[41]^.

Other non-specific presentations may include cardiac symptoms, such as dyspnoea, haemoptysis, and syncope, which can mimic other cardiac pathologies causing diagnostic uncertainty^[41]^. Furthermore, tumours can also be associated with other less frequently occurring cardiac symptoms, including conduction abnormalities, such as arrhythmias and heart blocks, comprising AV and complete heart block^[2,41]^. This results from either a mass effect on the conduction system of the heart or direct irritation of the myocardium^[3]^. Hence, although unlikely, cardiac tumours should be considered as a differential diagnosis in patients presenting with a new and sudden onset of such symptoms.

Cardiac metastasis can present with varied clinical manifestations depending on the site of metastatic invasion^[42]^. Tumours invading the myocardium may go unnoticed with generalised vague symptoms; whereas a more extensive invasion into the pericardium may result in acute medical emergencies, such as hemorrhagic pericardial effusions causing cardiac tamponade^[42]^. Occasionally, a neoplasm-induced embolism can result in myocardial infarctions or pulmonary embolisms due to occlusions of the coronary or pulmonary vasculature, respectively^[42]^. Lastly, presentations may be generalised and include symptoms such as fevers, arthralgia, anorexia, and weight loss^[42,43]^.

## DIAGNOSIS

The spectra of clinical manifestations include valvular obstruction, thromboembolism, arrhythmia, or pericardial disease. Systemic manifestations have been strongly implicated with the release of inflammatory mediators, such as interleukin-6^[3,44]^. Diagnosis is often delayed due to the varied, non-specific presentation. Extensive initial work-up with routine bloods, an electrocardiogram (ECG), and chest X-ray offers only non-diagnostic findings, hence clinical suspicion is necessary^[9]^. It remains that a small proportion of these tumours are subclinical and are identified incidentally during an investigation for other pathologies.

Imaging techniques are utilised when there is clinical suspicion of a disease and provide a more definitive diagnosis; common modalities include echocardiography, contrast-enhanced C-CT, C-MRI, and FDG-PET^[2,9,11]^. Echocardiography represents a diverse, noninvasive first-line imaging modality with high sensitivity and specificity (90% and 95%, respectively)^[9]^.

Echocardiography can aid quantification of the tumour’s site, size, and attachment, and combined with duplex ultrasound it can allow assessment of valvular disease and guide surgical planning^[4,9]^. Transthoracic echocardiography (TTE) used initially can help visualize ventricular masses, however, transoesophageal echocardiography (TOE) affords high-frequency transducers, better acoustic windows, and additional imaging planes, allowing superior visualization of the posterior chamber and atrial segments, as well as identification of small tumours (< 5 mm)^[6,9]^. The recent development of contrast echocardiography aids differentiation of malignant cardiac tumours from thrombi, with the former displaying rich vascularity and image enhancement with the administration of contrast medium^[9]^. Moreover, three-dimensional (3D) echocardiography provides improved structural assessment of tumours and relationship to adjacent structures. Limitations of echocardiography, however, include operator dependence, restricted fields of view, and difficulty in tumour subtype and tissue characterization with unfavourable body habitus^[2,6,40,45]^.

Further assessment with contrast-enhanced C-CT provides high-resolution imaging and accurate depiction of cardiac morphology and surrounding structures through the use of X-ray attenuation^[2,46]^. Moreover, technological advances in CT through use of multidetector computed tomography (MDCT), post-processing algorithms, and ECG gating have resulted in much more detailed imaging, delineation of smaller tumours, and evaluation of fat and calcification within studied masses^[6,9,47]^. Limitations of C-CT include exposure to significant radiation, lower temporal resolution, and poorer soft-tissue resolution compared to C-MRI^[6,9]^.

The use of C-MRI is increasingly becoming the imaging technique of choice for cardiac tumours as it allows the most favourable tissue characterization without ionizing radiation exposure^[6]^. C-MRI affords high spatial resolution views, multiplane assessment, and manipulatable unrestricted views, and it can be used in conjunction with enhancement through gadolinium administration, which aids particularly differentiating cardiac tumours from thrombi or flow artifacts^[6,9,48]^.

In addition, further developments in molecular imaging modalities, such as PET using 18F-FDG tracer, have greatly benefitted noninvasive visualization of malignant cardiac tumours and assessment of their metabolic activity^[7]^. FDG-PET particularly allows for exceptional accuracy in the staging of disseminated disease and prognostication^[7,9]^.

Table 4 is a summary of the imaging modalities that are useful in the diagnosis of cardiac tumours.

**Table 4:** Imaging techniques used in diagnosis of cardiac tumours.

Investigation	Benefit	Disadvantage	Specificity	Sensitivity
Echocardiography	• Noninvasive • TOE superior to TTE in identification of small tumours • Contrast/3D echo affords excellent structural assessment^[2,9,8]^	• Operator dependent • Difficulty in visualising masses with unfavourable body habitus • Does not allow tissue subtyping^[9]^	95%	90%^[9]^
C-CT	• Contrast-enhanced high-resolution imaging provides accurate morphological assessment differentiating benign from malignant tumours • Delineation of smaller tumours with MDCT and ECG gating techniques^[2,9,11]^	• Significant radiation exposure and nephrotoxicity of contrast • Poor soft-tissue evaluation	86%	82%^[9,11,45]^
C-MRI	• High-resolution, multiplane assessment • Nonionizing radiation • Tissue characterization based on signal patterns with weighted or contrast studies^[6,9,30]^	• Limited availability and costly to operate • Prolonged study compared to CT, may require anesthesia^[45]^	95%	90%^[6,30]^
FDG-PET	• Can be used to assess metabolic response to therapy • Excellent diagnostic evaluation preoperatively and for prognostication^[7,48]^	• High radiation exposure • Limited spatial resolution • Lack of availability and requires specialist interpretation^[7]^	86%	100%^[2,9,7]^

3D=three-dimensional; C-CT=cardiac computed tomography; C-MRI=cardiac magnetic resonance imaging; CT=computed tomography; ECG=electrocardiogram; FDG-PET=fluourodeoxyglucose-positron emission tomography; MDCT=multidetector computed tomography; TOE=transoesophageal echocardiography; TTE=transthoracic echocardiography

Histopathological evaluation of cardiac tumours for definitive classification and grading requires biopsy sampling^[9,40]^. Minimally invasive techniques are usually employed to minimize risks of cancer seedling and spread from a malignant primary tumour^[2,9,40]^. Common techniques include the cytological examination of pericardial or pleural fluid, echocardiographically-guided cardiac biopsy, or even thoracoscopically-guided biopsy^[9]^.

## MANAGEMENT

Cardiac tumours have the potential to lead to debilitating symptoms and haemodynamic dysfunction and compromise. Advancements in early diagnosis and management mean improved duration and quality of life in affected patients. Management of cardiac tumours should comprise a multidisciplinary team approach and evidence-based care depending on histopathological classification, cancer invasion, and patient risk stratification^[11]^.

### Medical

Conservative management of cardiac tumours is usually restricted to cases of small, immobile masses, such as papillary fibroelastomas, lipomas, and lipomatous hypertrophy, which typically progress slowly and are followed-up by serial echocardiography to monitor growth or development of symptoms^[2,4,10]^. In benign growths, the prognosis is generally very good, particularly in asymptomatic individuals^[9]^.

Malignant primary cardiac tumours infiltrate aggressively and carry poor prognosis with high rates of relapse^[49]^. The median survival of all malignant primary cardiac tumours is between 10-24 months^[4]^, with cited reasons being late presentation and difficult surgical management of locally advanced tumours. Management should ideally combine adjuvant chemoradiotherapy and complete surgical resection to reduce the risk of recurrence^[2,4,9]^. Studies have demonstrated that complete surgical resection alone improves median survival 3-fold compared to patients managed expectantly^[50]^.

Adjuvant chemotherapy administered early in the management of cardiac tumours has been shown to reduce the tumour’s size and improve duration and quality of life when used as an auxiliary to surgical resection or in palliation of unresectable disease^[9]^. Common treatment regimens include a combination of paclitaxel, doxorubicin, prednisone, cyclophosphamide, or vincristine and demonstrate a positive effect in most subtypes of malignant primary cardiac tumours, including cardiac lymphoma^[2-4,11]^.

Targeted radiotherapy used pre or postoperatively is an effective adjuvant to surgical resection for sarcomas, particularly low-dose radiotherapy^[2,9]^.

### Surgical

Advancements in cardiac bypass have been correlated with improved operative management of intracardiac masses. Benign primary cardiac tumours are primarily managed with surgical resection, providing relief from symptoms and allowing evaluation of tumour characteristics and prognostication^[4,9]^. Cardiac myxoma resection results in excellent long-term outcomes and low periprocedural mortality (< 5%)^[2]^. Follow-up is with a TOE at the postoperative first year and every five years thereafter^[2,9]^.

Similarly, the majority of papillary fibroelastomas undergo surgical resection, particularly in cases of large (> 10 mm) or mobile valve masses^[9]^, which may require robotic-assisted surgery over conventional sternotomy approaches, in which reconstruction of the mitral chordae is required^[2,9,51]^ if an extensive tumour invasion is present.

Rhabdomyomas tend to regress spontaneously with age, hence surgical management with subtotal excision is usually not indicated unless patients develop severe symptoms^[4]^. Rhabdomyomas have also been successfully managed using Everolimus, an mTOR complex 1 inhibitor^[2]^. Similarly, cases of fibromas, lipomas, and LHAS tumours only require surgical intervention when they cause haemodynamic compromise^[2,9]^.

Management of malignant primary cardiac tumours differs as adjuvant chemoradiotherapy is utilised to aid operative success. In the management of ventricular sarcomas, provided patients have an appropriate performance status, surgical resection with full or partial thickness excision should be attempted, with additional reinforcing with an endocardial patch to provide structural integrity postoperatively if necessary^[4,9]^. Angiosarcomas, the most common primary malignant tumours in adulthood, are aggressive tumours with a median survival as low as five months, compared to 17 months for other cardiac sarcomas. With isolated intracardiac masses, complete surgical resection is the treatment of choice, providing prognostic advantage^[4,9]^.

Primary cardiac lymphomas are extremely rare and associated with poor prognosis. Optimal management requires prompt diagnosis through histological evaluation, with the majority of patients expressing CD20+ diffuse large B-cell lymphoma^[2,9,30]^. Treatment of cardiac lymphoma is typically with Rituximab, a monoclonal antibody, used in conjunction with chemotherapy and even directed radiotherapy in some cases^[52,53]^.

## CONCLUSION

Cardiac tumours are rare, and yet are still a debilitating and possibly life-threatening condition. Technological advancements and increased availability of imaging modalities have enabled earlier and more accurate detection of these tumours. Moreover, a more detailed assessment through molecular mechanisms has resulted in better comprehension and management offered through tissue characterization and prognostication. Novel medical therapies, recommendations for screening, and operative techniques have all contributed to overall improving knowledge of these tumours and ultimately patient outcomes.

**Table:** 

Authors' roles & responsibilities	
MJ	Substantial contributions to the conception or design of the work; or the acquisition, analysis, or interpretation of data for the work; drafting the work or revising it critically for important intellectual content; agreement to be accountable for all aspects of the work in ensuring that questions related to the accuracy or integrity of any part of the work are appropriately investigated and resolved; final approval of the version to be published
SK	Substantial contributions to the conception or design of the work; or the acquisition, analysis, or interpretation of data for the work; drafting the work or revising it critically for important intellectual content; agreement to be accountable for all aspects of the work in ensuring that questions related to the accuracy or integrity of any part of the work are appropriately investigated and resolved; final approval of the version to be published
AN	Substantial contributions to the conception or design of the work; or the acquisition, analysis, or interpretation of data for the work; drafting the work or revising it critically for important intellectual content; agreement to be accountable for all aspects of the work in ensuring that questions related to the accuracy or integrity of any part of the work are appropriately investigated and resolved; final approval of the version to be published
AH	Substantial contributions to the conception or design of the work; or the acquisition, analysis, or interpretation of data for the work; drafting the work or revising it critically for important intellectual content; agreement to be accountable for all aspects of the work in ensuring that questions related to the accuracy or integrity of any part of the work are appropriately investigated and resolved; final approval of the version to be published
